# Nitric oxide participates in plant flowering repression by ascorbate

**DOI:** 10.1038/srep35246

**Published:** 2016-10-12

**Authors:** Rajendran Senthil Kumar, Chin-Hui Shen, Pei-Yin Wu, Subbiah Suresh Kumar, Moda Sang Hua, Kai-Wun Yeh

**Affiliations:** 1 Institute of Plant Biology, National Taiwan University, Taipei 106, Taiwan

## Abstract

In *Oncidium*, redox homeostasis involved in flowering is mainly due to ascorbic acid (AsA). Here, we discovered that *Oncidium* floral repression is caused by an increase in AsA-mediated NO levels, which is directed by the enzymatic activities of nitrate reductase (NaR) and nitrite reducatase (NiR). Through Solexa transcriptomic analysis of two libraries, ‘pseudobulb with inflorescent bud’ (PIB) and ‘pseudobulb with axillary bud’ (PAB), we identified differentially expressed genes related to NO metabolism. Subsequently, we showed a significant reduction of NaR enzymatic activities and NO levels during bolting and blooming stage, suggesting that NO controlled the phase transition and flowering process. Applying AsA to *Oncidium* PLB (protocorm-like bodies) significantly elevated the NO content and enzyme activities. Application of sodium nitroprusside (-NO donor) on *Arabidopsis vtc*1 mutant caused late flowering and expression level of flowering-associated genes (*CO*, *FT* and *LFY*) were reduced, suggesting NO signaling is vital for flowering repression. Conversely, the flowering time of *noa*1, an *Arabidopsis* NO-deficient mutant, was not altered after treatment with L-galacturonate, a precursor of AsA, suggesting AsA is required for NO-biosynthesis involved in the NO-mediated flowering-repression pathway. Altogether, *Oncidium* bolting is tightly regulated by AsA-mediated NO level and downregulation of transcriptional levels of NO metabolism genes.

Flowering is a complicated process coordinated by environmental and endogenous factors to ensure plant reproduction in appropriate conditions. Forward and reverse genetic tools have shown the critical role of genes in photoperiodism (responding to low temperatures), aging and phytohormones in the regulation of flowering^1^. Noteworthy, current evidence has suggested that several antioxidants, such as ascorbate (AsA) and glutathione, function as negative repressors of flowering time^2^,^3^,^4^,^5^,^6^. The AsA-mediated flowering time can be assessed by the following two aspects: AsA level and redox ratio. *Arabidopsis VTC*1 encodes a GDP-mannose pyrophosphatase gene in a Smirnoff-Wheeler pathway for AsA biosynthesis. The *vtc1* mutant is deficient in AsA levels with 40% of the AsA amount of wild-type(wt) plants, and it displays facilitated flowering under a long-day photoperiod^7^,^8^. Other AsA-deficient mutants encoding different genes in the Smirnoff-Wheeler pathway displayed early flowering similar to that of *vtc1*^5^. Furthermore, the expression levels of genes participating in the photoperiodic flowering pathway, including *FT*, and *CO*, are promoted in the AsA-deficient mutants. Various photoperiodic and autonomous pathway mutants are epistatic to *vtc1*, the flowering time of the *vtc1* mutant growing under a short-day photoperiod is susceptible to light intensity. The endogenous AsA level prominently declines when the plants are in transition from the vegetative stage to the reproductive stage accompanied with an elevated expression level of *OgLEAFY*^6^. In contrast, exogenous application of AsA or its precursor causes delayed flowering of *Brassica rapa*, *Arabidopsis* and *Oncidium*^6^,^9^,^10^. This evidence suggests that the effect of AsA on the repression of flowering is flexible and rapid in response to environmental cues. AsA functions as a co-factor in numerous phytohormone biosynthesis pathways and regulates endogenous levels of gibberellin, abscisic acid, salicylic acid, and ethylene^4^. Therefore, the effect of AsA on flowering has been speculated to alter phytohormone levels. AsA-mediated flowering is primarily proposed in a mechanism independent of its antioxidant activity because of an invariable H_2_O_2_ level in the *vtc1* mutant compared to wild type^5^. However, other reports have shown that H_2_O_2_ level increases before floral initiation of morning glory (*Pharbitis nil*)^11^, *wheat*^12^, *Arabidopsis*^13^ and *Oncidium*^6^. In addition, enervated flowering is present in several mutants lacking enzymes that oxidize AsA to dehydroascorbate (DHA), including APX and AsA oxidase^14^,^15^. Additionally, the negative role of NO on floral induction in *Arabidopsis* has been validated^16^. The *nox1* mutant disrupts a chloroplast phosphoenolpyruvate/phosphate translocator to accumulate L-arginine at a higher level than wild type, thus exhibiting higher NO emission and delayed flowering^16^,^17^. NO produced from the nitrate-related system displays a 100-fold greater output than NO produced from an arginine-associated or NOS-like system, which demonstrates the crucial role for nitrate reductase (NaR) in NO synthesis in *planta*^18^,^19^. AsA is an important co-factor for NOS activity in mammals^20^, but the genes encoding mammal-like NOS in plants are still unidentified^21^.

The life cycle of *Oncidium* ‘Grower Ramsay’ starts off with its vegetative stage and may progress into two different life pathways as follows: either flowering with inflorescence (transition to reproductive phase) or regenerating a new axillary bud (retaining the vegetative stage) (Fig. 1a). The determining factors for these two phase-transitions are still unknown. Previously, we have demonstrated that endogenous AsA is essential for phase transition and the flowering process^6^. Similarly, the redox homeostasis of *Oncidium* is reliant on ambient temperature as well as phase transition signaled by the decrease in AsA levels in ‘pseudobulb with inflorescent bud’ (PIB) tissues^22^,^23^. However, the specific mechanism of AsA and NO signaling in repressing flowering is poorly understood. In the present study, we demonstrated that the repression of *Oncidium* flowering is determined by the coordinated action of ascorbic acid and nitric oxide. We presented evidences that the early flowering phenotype of *Arabidopsis vtc*1 mutant line was delayed after exogenous sodium nitroprusside (SNP) application. Our data also showed that the biochemical activities of NO-biosynthesis (NiR and NaR) in *Arabidopsis vtc*1 mutant lines were obviously lower than those of wild type. In contrast, the early flowering time of *noa1*, a NO accumulation-deficient mutant, was not altered after treatment with L-galacturonate, a precursor of AsA production. Altogether, we showed that the flowering repression mechanism is mainly dependent on NO level, which is mediated by AsA content through regulating the NO-biosynthetic enzymatic activity.

## Results

### Solexa sequencing: Statistical characterization of global gene expression

**Figure 1:**
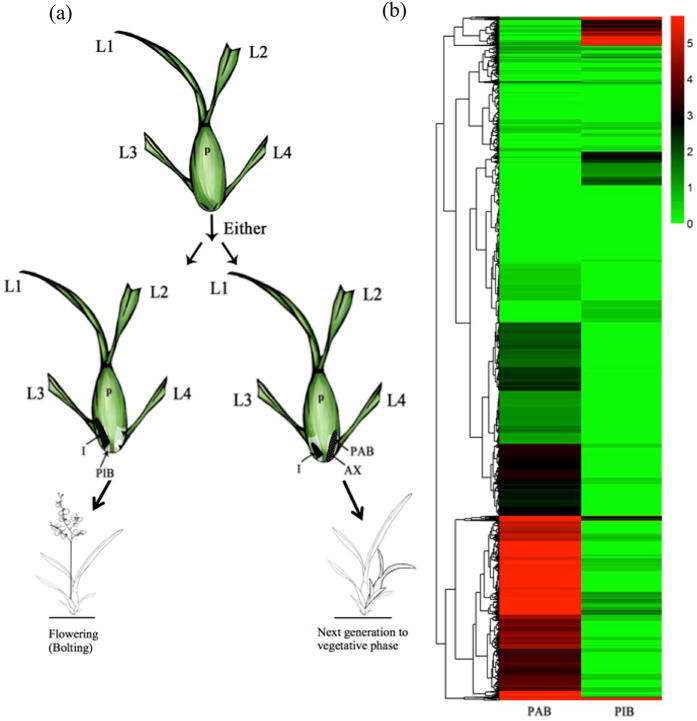
Tissue-specific sorting of *Oncidium* transcriptomes. **(a)** Diagram of the *Oncidium* plant life cycle. The inflorescent bud (I) and axillary bud (AX) are formed concomitantly at each side of the pseudobulb (P) base at the vegetative stage. Either bolting or developing to next generation is regulated by a complicated genetic network (modified based upon previously published^6^,^22^). L. denotes the leaf numbering from the top to the base. (**b**) Heat map showing unsupervised hierarchical clustering of differential expression genes between PAB and PIB with p value <0.5 and fold change ≥5.0. Red indicates a high expression level, and green indicates a low expression level. White indicates no expression. PAB: pseudobulb with axillary bud. PIB: pseudobulb with inflorescent bud.

Solexa deep sequencing technology was performed to sequence the transcriptome of ‘pseudobulb with inflorescent bud’ (PIB) and ‘pseudobulb with axiliary bud’ (PAB) (Fig. 1a). After trimming adapter sequences and removing sequences shorter than 75 bases, sequencing depths of 925,937 and 665,127 contigs were achieved in PAB and PIB libraries (Supplementary Table S1) with a total of 106.1 million and 79.3 million reads, respectively. The most-aligned results displayed a total of 51,883 (47.8%) and 32,747 (30.2%) afresh-assembled unigenes, which were annotated in this manner by Nr and Swiss-Port respectively and oriented for subsequent analysis (Supplementary Table S1). The expression levels of the assembled unigenes indicated that 98,711 (90.9%) unigenes displayed similar or extremely low expression levels between the two libraries (Fig. 1b). The parallel majorities within the two categories in the PAB and PIB libraries were as follows: metabolic process and cellular process in biological processes; catalytic activity and binding in molecular functions; and organelle and cell in cellular components (Supplementary Fig. S1). Supplementary Table S2 illustrates the functional enrichment analysis of PAB library versus PIB library was done by fatiGO executed in blast2go. The most significantly identified genes were related to ribosome biogenesis (GO: 0042254), chromatin assembly or disassembly (GO: 0006333) and cell redox homeostasis (GO: 0045454) within cellular process subcategory (GO: 0009987), gluconeogenesis (GO:0006094) and ATP biosynthetic process (GO: 0006754) in metabolic process subcategory (GO: 0008152), GTPase activity (GO: 0003924) and hydrogen ion transmembrane transporter activity (GO: 0015078) within catalytic activity subcategory (GO: 0003824), GTP binding (GO: 0005525), peptide binding (GO: 0042277), magnesium ion binding (GO: 0000287), nucleic acid binding (GO: 0003676) and chromatin binding (GO: 0003682) within ‘binding’ subcategory (GO: 0005488), mitochondrial inner membrane (GO: 0005743) within ‘organelle’ subcategory (GO: 0044422) and ribosomal subunit (GO: 0033279) within ‘cell’ subcategory (GO: 00044464) (Supplementary Table S2, Supplementary Fig. S2). The majority of the mRNA transcripts from the PAB library was proposed to function in promoting next generation vegetative growth or floral repression. The genes participating in NOS orthologue proteins, GTP binding (GO: 0005525) and magnesium ion binding (GO: 0000287), were absent in the PIB libraries of inflorescence initiation (Supplementary Table S2, Supplementary Fig. S2).

### Differential expression of NO- biosynthesis- related genes correlated with phase transition

**Figure 2:**
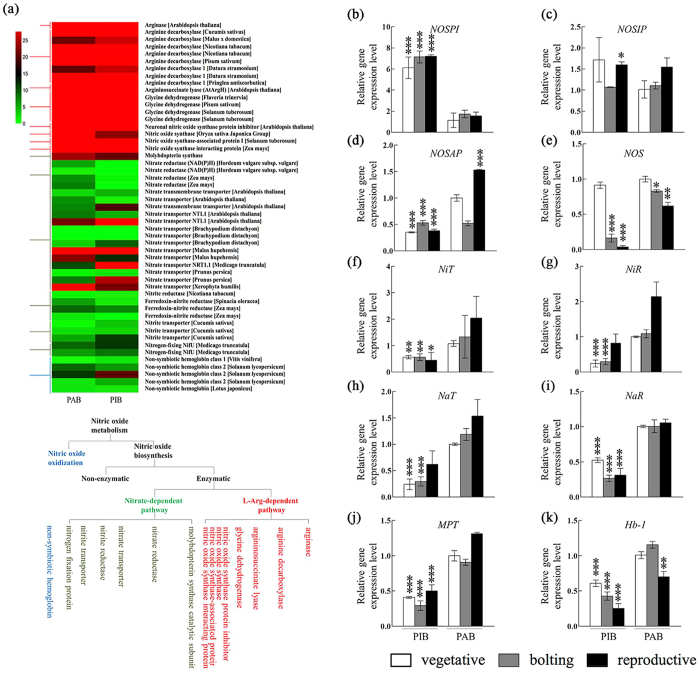
The expression pattern of 51 assembled unigenes involved in nitric oxide metabolism from two libraries. **(a)** The pathway of NO metabolism in *planta*. The heatmap of expression profiling generated from two transcriptomic libraries. Red indicates a high expression level, and green indicates a low expression level. PAB: pseudobulb with axillary bud. PIB: pseudobulb with inflorescent bud. **(b–k)** The relative gene expression levels of the following ten genes involved in NO metabolism were monitored in PIB and PAB at the vegetative stage (□ white square), the bolting period (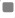 gray square) and the reproductive stage (■ black square): NOS-interacting protein (*NOSIP*); NOS protein inhibitor (*NOSPI*); NOS-associated protein (*NOSAP*); nitric oxide synthase-like (NOS); nitrite transporter (*NiT*); nitrite reductase (*NiR*); nitrate reductase (*NaR*); nitrate transporter (*NaT*); molybdopterin synthase catalytic subunit (*MPT*) and non-symbiotic hemoglobin (*Hb-1*). Gene expression levels in each sample were compared to those in PAB at the vegetative stage. Error bars indicate standard deviation (s.d.). Asterisks represent significant differences from PAB tissue at the vegetative stage according to two-way ANOVA (*p < 0.05; **p < 0.005; ***p < 0.0005).

The functional enrichment analysis of two transcriptomic profiles from PAB and PIB tissues were investigated. The data showed that nitrogen-associated metabolism was active in PAB and not in PIB. The genes were grouped into three clusters, namely the L-arginine-dependent pathway, the nitrite-dependent pathway and the class II non-symbiotic hemoglobin pathway (Fig. 2a). The genes participating in the L-arginine-dependent pathway displayed a consistently high expression level, while the genes in the nitrate-dependent pathway and non-symbiotic hemoglobin displayed relatively low expression levels (Fig. 2a). Genes participating in the L-arginine-dependent pathway showed little difference between the two transcriptomic libraries and they presented higher signal intensity. To further verify the Solexa analysis results, the expression levels of ten NO metabolism genes were monitored by RT-PCR (Fig. 2b–k). Nitrate reductase (NaR), nitrite reductase (NiR) and among others, displayed significantly reduced expression levels in PIB compared to those in PAB (Fig. 2d–k), similar to the patterns in heat map (Fig. 2a). In contrast, the NOS protein inhibitor (NOSPI) and NOS interacting protein (NOSIP) exhibited higher expression levels in PIB than in PAB (Fig. 2).

### Low NO level was associated with low NaR activities and repressing phase transition and floral initiation of *Oncidium*

**Figure 3:**
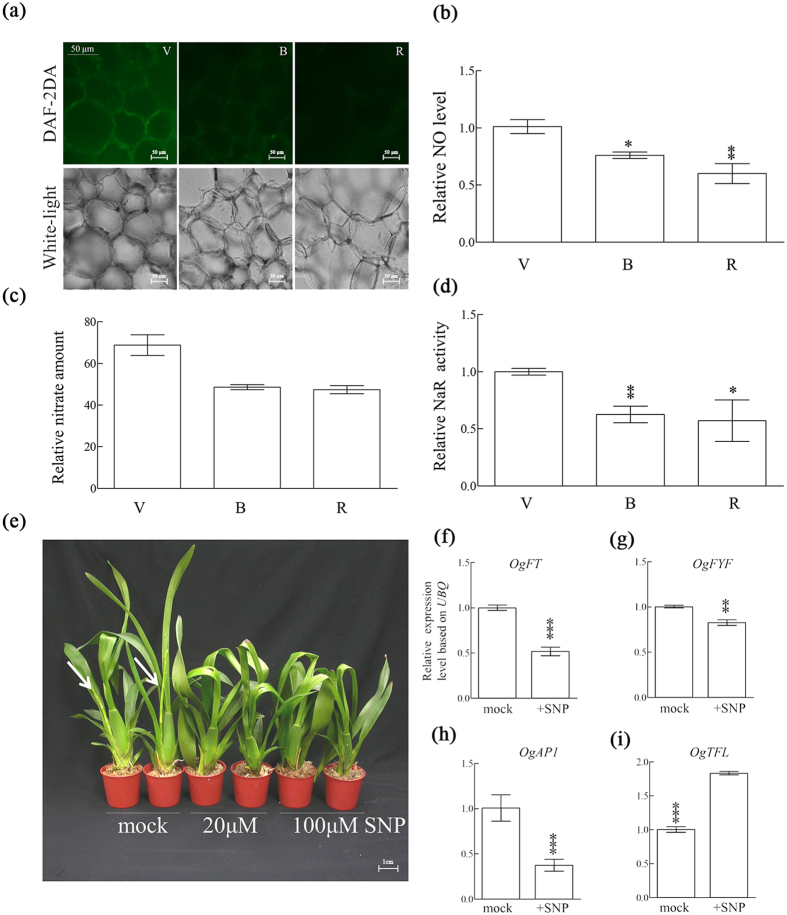
The activities of NO biosynthetic enzymes and the levels of NO in *Oncidium* PIB tissues at different stages. (**a)** The NO intensity was detected using the green fluorescence of DAF-2T, and white light indicated the morphology and cell status in PIB tissues(scale bar = 50μM) (**b**–**d**) The nitric oxide (NO) levels (b), nitrate level (c) and nitrate reductase (NaR) enzymatic activity (d) were quantified in *Oncidium* PIB tissues at the vegetative (V), bolting (B) and reproductive (R) stages. **(e)** Photographs of *Oncidium* orchids treated with water (mock), 20 μM SNP or 100 μM SNP, which was applied on the juvenile inflorescent buds for one month. White arrows indicate the developing inflorescence (scale bar = 1cm). (**f–i)** Expression levels of floral genes in the inflorescence of mock or SNP-treated *Oncidium* orchids. The relative gene expression levels in SNP-treated plants were compared to those of mock plants. Error bars indicate standard deviation (s.d.). Asterisks represent significant differences compared with vegetative stage or mock respectively and were analyzed by two-way ANOVA (*p < 0.05; **p < 0.005; ***p < 0.0005).

To analyze the endogenous NO level, microscopic observation with the aid of the NO-specific fluorescent dye, 4, 5-diaminofluorescein diacetate (DAF-2DA), revealed that the NO level at the reproductive stage was decreased by approximately 60% compared to that of the vegetative stage (Fig. 3a). To further investigate NO production, we assayed the NaR activity and quantified NO level and nitrate concentration in *Oncidium* at the following three different stages: vegetative (V), bolting (B), and reproductive (R). Our data demonstrated that the NO level, nitrate concentration and NaR enzymatic activity were markedly decreased in the period between the vegetative stage and the reproductive stage (Fig. 3b–d), suggesting a low production of NO. Orchids at the vegetative stage were subjected to different concentrations of SNP, an effective NO donor functionally known to repress floral transition in *Arabidopsis*. Floral transition was repressed after treatment with 20 and 100 *μ*M SNP (Fig. 3e). These results clearly indicated that the NO molecule is an effector that can repress floral initiation in *Oncidium*. Furthermore, we analyzed the following flowering-associated genes by qRT-PCR: *OgFT*, *OgFYF*, *OgAP1*, and *OgTFL* (Fig. 3f–i). Our results showed that the transcript levels of *OgFT*, *OgFYF*, and *OgAP1* were lowered, but the transcript levels of *OgTFL,* a floral repressor, were enhanced by SNP treatment (Fig. 3f–i).

### NO level and the activity of NaR-related NO biosynthesis pathway were enhanced by ascorbate

**Figure 4:**
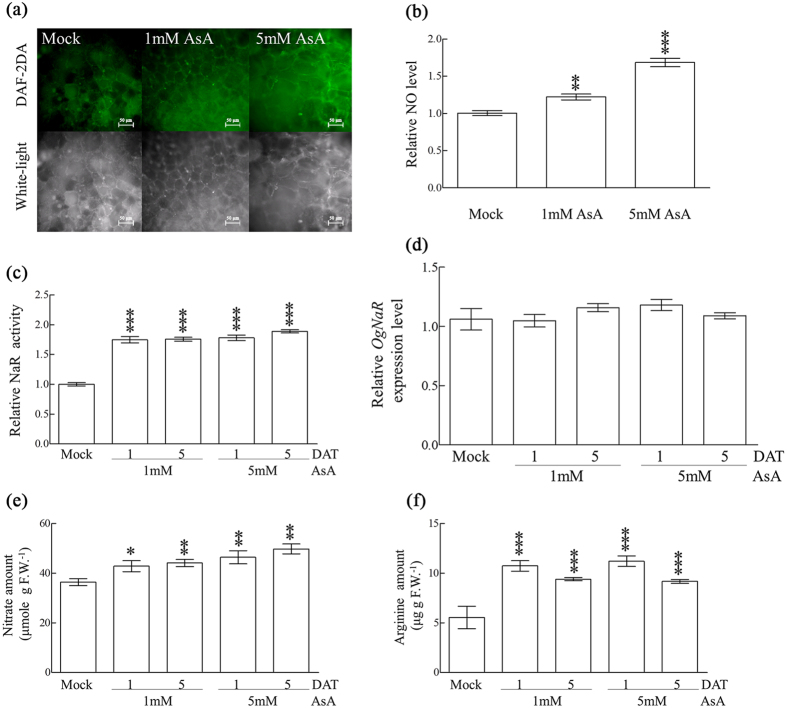
NO-related metabolism in *Oncidium* protocorm-like body (PLB) tissues treated with ascorbate (AsA). (**a)** The NO intensity of *Oncidium* PLB tissues grown in ½ MS without treatment (mock), or under treatment with 1 mM and 5 mM AsA for 5 days. NO intensity was indicated by a green fluorescence of DAF-2T, and white light indicated the morphology and cell status in tissues (scale bar = 50μm). **(b)** The relative NO intensity in AsA-treated *Oncidium* PLB tissues was compared to that in mock *Oncidium* PLB cultures. **(c,d)** The relative activities of nitrate reductase (NaR) and *OgNaR* gene expression in AsA-treated *Oncidium* PLB, comparing to those of mock *Oncidium*. **(e**,**f**) Nitrate and arginine amounts in mock or AsA-treated *Oncidium* PLB tissues are shown. Significant differences compared with mock were analyzed by two-way ANOVA (*p < 0.05; **p < 0.005; ***p < 0.0005).

It is well-known that the reduced form of ascorbate (AsA) and reactive oxygen species are essential for phase transition in *Oncidium*^6^,^22^,^23^. To further elucidate the relationship of AsA and NO during phase transition of *Oncidium, Oncidium* tissue culture of a protocorm-like body (PLB) was used to monitor the effect of AsA on NO production. As shown in Fig. 4a the fluorescent intensity of *Oncidium* PLB, which indicated NO levels, increased under high doses (5 mM) of AsA compared to low doses of AsA (1 mM) and mock treatment. Accordingly, the NO levels and NaR enzymatic activities were elevated in response to exogenous AsA treatment (Fig. 4b,c). However, the transcript level of NaR was unaltered in response to AsA (Fig. 4d). In addition, the nitrate and arginine levels in *Oncidium* PLB tissues increased with AsA dosage (Fig. 4e,f). These results demonstrated that AsA is effective in elevating the precursor levels and enzyme activities for NO biosynthesis, thus increasing NO levels (Fig. 4).

### The AsA-deficient mutant, *vtc1*, produces low NO, leading to early flowering

**Figure 5:**
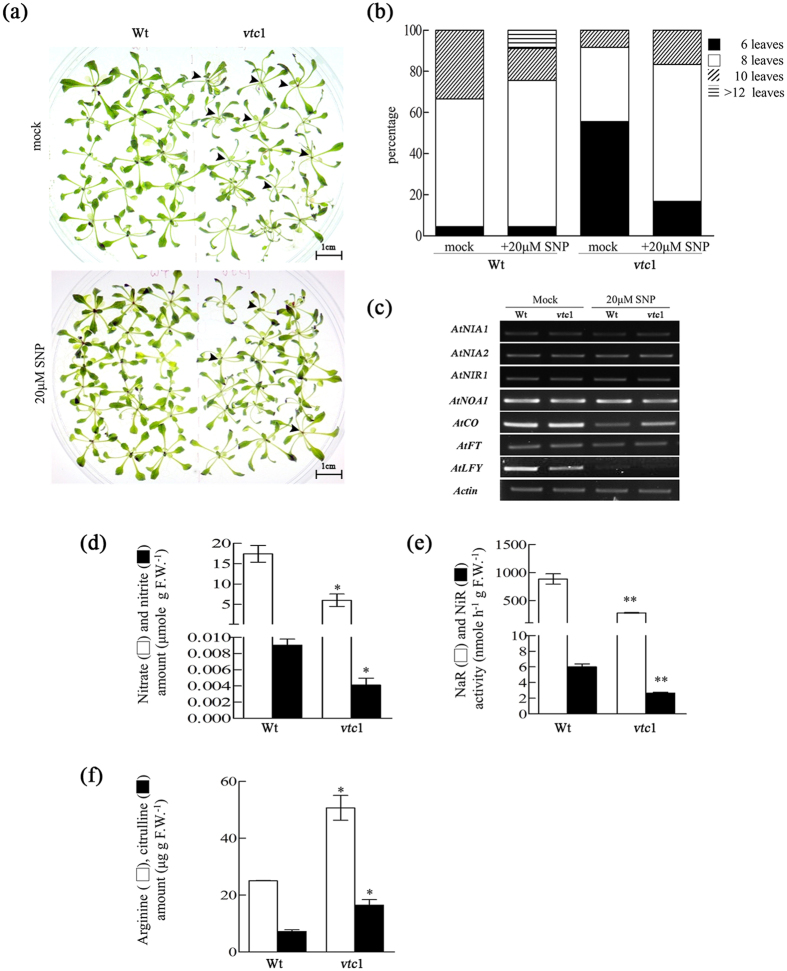
Endogenous NO and related compounds were analyzed in *A. thaliana* Col-0 (Wt) and *vtc1.* **(a)** Photographs of the Wt and *vtc1* mutant grown on the ½ MS medium under mock or 20 μM SNP for 3 weeks. It showed that *vtc1* showed earlier flowering than Wt (upper panel arrow marks), but 20 μM SNP decreased percentage of early flowering plants (lower panel) scale bar = 1cm). **(b)** The percentage of flowering Wt or *vtc1* at different development stages specified according to rosette leaf number under mock or SNP treatments (n _wt_ and n_*vtc1*_ ≥ 30). **(c)** RT-PCR analysis showed the expression pattern of NO-related and flowering-associated genes (*AtNIA1, AtNIA2, AtNIR1, AtNOA1, AtCO*, *AtFT*, and *AtLFY*) after treatment with 20 μM SNP (actin was used as an internal control). **(d)** The levels of nitrate (□) and nitrite (■) in the Wt and *vtc1* mutant. **(e)** The NaR activity (□) and NiR activity (■) in the Wt and *vtc1* mutant. **(f)** The levels of arginine (□) and citrulline (■) in the Wt and *vtc1* mutant. Error bar represents ± SE. Significant differences between samples were determined using two-way *ANOVA*.

To further validate the functional effects of AsA on NO production, *Arabidopsis vtc1* mutant was employed. The *Arabidopsis vtc1* mutant, containing 40% of the wild type AsA level and displaying an early flowering phenotype, was shown to have low NO level (Fig. 5a,b). On contrary, there were 80% of 20 μM SNP treated-*Arabidopsis vtc1*mutant lines flowered with 8 rosette leaves as compared to the mock-treated *Arabidopsis vtc1* mutant lines (Fig. 5b). A comparative survey of the NO- biosynthesis-related genes, such as *AtNIA1* (AT1G77760), *AtNIA2* (AT1G37130), *AtNIR1* (AT2G15620) and *AtNOS/AtNOA1* (AT3G47450), revealed similar expression levels after SNP treatment between the two plants (Fig. 5c, Supplementary Fig. S3). This indicated that SNP did not affect transcription activity of NO- biosynthesis-related genes. Furthermore, the expression patterns of flowering-associated genes, namely *CONSTANS* (*CO)*, *GIGANTEA* (*GI)*, *Flowering locus T (FT)* and *LEAFY* (*LFY),* were assayed in plants treated with SNP. The transcript levels of *CO*, *GI*, *FT* and *LFY* were lowered by SNP treatment (Fig. 5c). However, lower levels of nitrate and nitrite were discovered in *vtc1* accompanied by decreased NaR and NiR enzymatic activities (Fig. 5d,e). In contrast, *Arabidopsi*s *vtc1* contained higher levels of arginine and citrulline than the wild type (Fig. 5f). No significant differences in the expression of NO- biosynthesis-related genes were observed between in Wt and *vtc1* mutant. However, the levels of nitrate and nitrite, activities of NaR and NiR were lower in *vtc*1 due to its lower AsA level, suggesting that lower AsA level caused lower level of NO production and led to early flowering.

**Figure 6:**
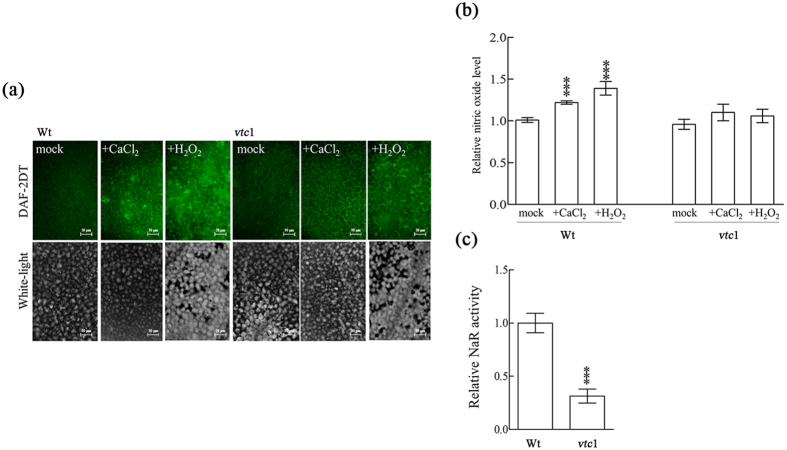
CaCl_2_ and H_2_O_2_ were ineffective to induce NO in *Arabidopsis vtc*1 mutant line. **(a)** The NO intensity in leaf segments of the Wt and *vtc1* mutant treated with water (mock), CaCl_2_ and H_2_O_2_. White light indicated the morphology and cell status in tissues (scale bar = 50μm). **(b)** The relative NO intensity in treated Wt and *vtc1* was compared to that of mock Wt. **(c)** The relative activities of nitrate reductase (NaR) after treatment of CaCl_2_ or H_2_O_2_ in the *vtc1* mutant, were compared to those of Wt.

CaCl_2_ and H_2_O_2_ have been reported to act as elicitor to induce NO production. However, our results showed that CaCl_2_ and H_2_O_2_ were effective to induce NO production in wild type, whereas both were ineffective to induce NO production in *vtc1* (as shown by florescent intensity) (Fig. 6a). Additionally, compared to the wild type, *Arabidopsis vtc*1 mutant line displayed moderately lower production of NO in response to CaCl_2_ and H_2_O_2_ (Fig. 6b). It was further confirmed through enzymatic assay of NaR, that NO production was significantly lowered in the *Arabidopsis vtc1* mutant line compared to the wild type (Fig. 6c). These data suggested that change in AsA redox ratio in *Arabidopsis vtc*1 line had no direct influence on endogenous NO level.

### The effect of L-galactonolactone (L-GalL) on the flowering time of *A. thaliana* Col-0 (Wt) and *noa1*

**Figure 7:**
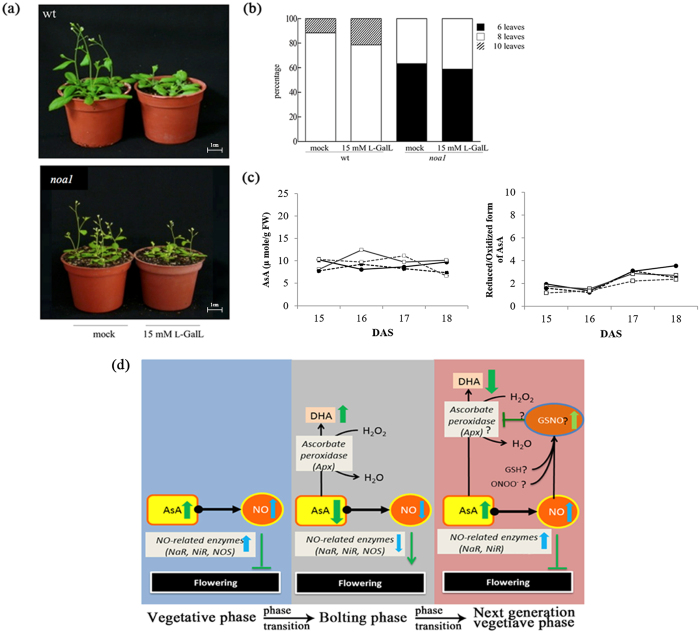
Flowering time change of *A. thaliana* Col-0 (Wt), and *noa1* mutant in response to exogenous L-galactonolactone (L-GalL) treatment. (**a**) The effect of L-galactonolactone (L-GalL) on the flowering time of *A. thaliana* Col-0 (Wt) and *noa1* (scale bar = 1cm). (**b**) The percentage of flowering Wt or *noa1* at different development stages specified according to rosette leaf number under mock or L-GalL treatments (n _wt_ and n_*noa1*_ ≥30). Error bars indicate standard deviation (s.d.). Asterisks represent significant differences between samples as determined using two-way *ANOVA* (Wt; *p < 0.05; **p < 0.005; ***p < 0.0005). (**c**) The ascorbic content and ratio of reduced/oxidized form of AsA in *A. thaliana* Col-0 (Wt) and *noa1* grown in ½ MS without treatment (mock: (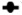), Wt; (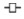), *noa*l mutant), or under treatment with 20 μM SNP (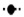), Wt; (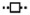), *noa*1 mutant) for 3 weeks. Significant differences compared with mock were analyzed by two-way ANOVA (*p < 0.05; **p < 0.005; ***p < 0.0005). (**d**) Working model of the *Oncidium* floral repression acquired by post-translational modification of NO- biosynthesis-related enzymes: During the vegetative stage, the AsA level, NO-related enzymes (NaR, NiR and NOS) and NO levels were high and synergistically repressed flowering. In the bolting stage, DHA was produced by the redox homeostasis of *Oncidium*, which lowered the AsA redox ratio, thus leading to deceased NO-related enzymatic activities for NO and phase transition. In the next vegetative stage, low levels of AsA and NO were triggered by the redox homeostasis, thus leading to the accumulation of GSNO to terminate the reproductive stage through floral repression and post-translational modification of APX.

To ascertain AsA-mediated function in NO biosynthesis and the repressive role in the flowering process, exogenous L-GalL, a precursor of AsA, was applied on *Arabidopsis* Wt and *Arabidopsis noa1* mutant, a NO-deficient mutant and displayed early flowering. As shown in the Fig. 7a, the wild type plants showed delayed flowering after L-galactonolactone (L-GalL) treatment. Comparing to the mock treatment, *Arabidopsis noa1* displayed slightly delayed flowering time with 6–8 rosette leaves after L-galactonolactone (L-GalL) treatment (Fig. 7a,b). To further investigate the relationship of AsA on NO metabolism, we assayed total AsA content and the redox ratio of reduced/oxidized form of AsA in *Arabidopsis* Wt and *noa1* mutant under SNP treatment. As shown in the Fig. 7c, the level of AsA showed an increase in *noa1* mutant lines compared with Wt. This result indicated that AsA is accumulated in *noa1* mutant regardless it’s low NO accumulation and early flowering phenotype, compared to the wild type. On the other hand, Wt *Arabidopsis* seedling treated with SNP significantly reduced in endogenous AsA level. Moreover, the redox ratio of reduced/oxidized form of AsA was found to be lowered in *Arabidopsis noa1* mutant seedlings after SNP treatment (Fig. 7c). These results suggest that the flowering repression in *Arabidopsis* was determined by NO level, not the AsA content.

## Discussion

The genetic network of *Oncidium* that controls the flowering process and flowering time is quite complicated and not well understood. Large-scale sequencing approaches have been performed to reveal the flowering mechanism of *Oncidium*^24^,^25^,^26^. Our transcriptomic data revealed that NO-related genes were abundantly and differentially expressed in the hemisphere of pseudobulb tissue proximal to the inflorescence bud (PIB), compared to the other half proximal to the axillary bud (PAB) (Fig. 1). It is well known that the NO synthesis can occur both enzymatically and non-enzymatically in plants^18^,^27^,^28^. However, the source of NO production in plants has not yet been identified and is hotly debated^29^. In mammals, NOS enzymes are key enzymes that catalyze L-arginine to generate NO and citrulline by NADPH-dependent oxidation in a complex reaction involving Ca^2+^, FMD, and CaM^30^,^31^. Similar mechanisms have also been reported in plants for the synthesis of NO from L-arginine and from nitrite^32^.

Our study also discovered that the mechanism that lowers NO production and reduces NaR and NO level was associated with *Oncidium* phase transition and repression of the flowering process (Fig. 3). The reduced stalk inflorescence and lower transcript levels of flowering-associated genes (*FYF*, *FT* and *AP1*) in *Oncidium* further confirmed that NO is a repressor of floral transition and the flowering process (Fig. 3)^16^. This study provided evidence that the level of nitrate, nitric oxide and arginine were enhanced in the presence of AsA (Fig. 4a,b,e,f). Although AsA is known to be involved in cellular redox signaling but does not act specifically in any of the known flowering pathways, it plays a general role in responding to environmental signals^5^. The reduced form of AsA is essential for *Oncidium* phase transition and flowering process under high ambient temperature^6^,^22^,^23^. A plausible explanation may be plants would rather produce NO than H_2_O_2_ to balance redox homeostasis for its physiological functions, that are tampered by AsA (Fig. 4)^33^,^34^. However, increased enzymatic activity of NaR is not dosage -dependent that could support the alternative pathway for NO production from direct reaction of AsA (Fig. 4c)^35^.

Based on recent publications, it was stated that NO production is directly proportional to AsA, either by modulating electron flow through the NaR enzyme or by indirectly affecting nitrite concentration, under strict regulation^36^,^37^. Therefore, we hypothesize that the posttranslational modification of *cyt*APX1 by NaR- dependent NO production, might contribute to floral repression^35^,^36^,^37^. The ascorbate-deficient mutant, *Arabidopsis vtc1,* displayed a lower level of nitrate and nitrite, as well as decreased NaR and NiR activities, compared to Wt (Fig. 5). This may explain by fact that higher level of reduced ascorbate doesn’t react with NO and its enzymatic activities but may be with other nitrosating species (Fig. 5)^34^,^35^,^36^,^37^,^38^.

Moreover, *Arabidopsis vtc1* had delayed flowering and decreased mRNA transcripts of flowering-associated genes (*CO*, *GI*, *FT* and *LFY*) after SNP treatment, implying that the delay flowering was due to the undesired side effects caused by SNP. In recent reports, NO donors (SNP, SNAP and SIN-1) induced flowering under non-inductive conditions in duckweed (*Lemma aequinoctialis*)^39^. However, SA-induced flowering in duckweed was significantly reduced by exogenous application of NO scavengers, nitric oxide synthase inhibitors and nitrate reductase inhibitor in duckweed, providing that NO was not involved in photoperiodic flowering pathway and acting as a stress negating agents from the SA-signaling pathway^40^. NO donor doesn’t necessarily mimic the functions of NO when level of reduced AsA is higher, but could rather involve in controlling the cellular redox signaling^41^,^42^. Note that, *Arabidopsis vtc1* mutant displayed increased level of arginine and citrulline level was significant compared with Wt (Fig. 5). It is speculated that AsA act as a redox cofactor for NOS to catalyze the conversion of arginine/citrulline for NO production^28^,^43^. However, this hypothesis is less well understood and requires extensive investigation. This evidence from Arabidopsis *vtc*1 supports a notion that the relationship between AsA and NO adds some degree of specificity as follows: (1) low AsA caused low level of NO production and leads to early flowering, (2) NO, being an inducer of antioxidant buffering system can alter antioxidant gene expression (*cyt*APX1) by ROS–dependent post-translation modification, (3) basal NO production depends on AsA and requires L-arginine and citrulline as supplements under oxidative stress^43^,^44^,^45^. Thus, the repression of flowering was determined by NO through NO-related enzymatic activities in the presence of AsA.

The *Arabidopsis vtc1* mutant line did not respond to hydrogen peroxide (H_2_O_2_) and calcium chloride (CaCl_2_) compared to wild type (Wt), suggesting the role of *cyt*APX1 maintained a low AsA ratio to scavenge H_2_O_2_ (Fig. 6)^5^,^6^,^22^,^23^. Molecular studies of the *Arabidopsis noa1* mutant further showed that AsA could not rescue flowering time (Fig. 7). Here, we proposed the function of AsA in NO biosynthesis is acting as a cofactor for NO mediated flowering repression process^46^,^47^. The *Oncidium* flowering process was strictly controlled by the mechanism involved in the signaling between AsA and NO biosynthesis with various degrees of specificity as follows: (1) *Oncidium* floral repression is dependent upon NO production, (2) NO biosynthetic enzymatic activities are repressed during bolting period of *Oncidium* PIB, (3) AsA-mediated NO signaling increases NO biosynthesis and enzymatic activities, which could activate post-translational modification for flowering repression, (4) low NO derivatives prevents the scavenging of excess free radicals that accelerate flowering in *Oncidium*, and they are maintained through redox reaction utilizing arginine as a substrate, (5) AsA and NO both play mutual role in flowering repression by inducing NaR–mediated and L-arginine dependent NO biosynthesis^48^.

In the immediate future, it is better to understand the molecular mechanism of NO production through redox signaling (ascorbate-glutathione cycle) from NaR–mediated and L-arginine-dependent (NOS-like) post-translational modification (APX), which may be useful to clarify the importance of antioxidants and their role in the repression of the flowering process by NO in plants (Fig. 7d)^49^.

## Materials and Methods

### Plants materials and growth conditions

*Oncidium* ‘Gower Ramsey’ plants were obtained from the Shih-Dong Orchid Nursery in Taiwan. The plants were grown in 30 cm diameter pots under growth conditions of 25~32 °C and a 14 h/10 h (day/night) photoperiod in a greenhouse. *Oncidium* protocorm-like bodies (PLBs), *Arabidopsis thaliana* ecotype Col-0, *vtc1* mutant (ABRC stock No.: CS8326) and *noa1* (ABRC stock No: CS6511) were cultured in 1/2 Murashige and Skoog medium^50^ under long-day conditions (16 h light/8 h dark cycles) at 23 ± 2 °C.

### RNA isolation and sequencing

Total RNA from each sample was isolated with TRIzol (Invitrogen) according to the manufacturer’s instructions. After being treated with RNase-free DNase I (New England BioLabs) for 30 min at 37 °C to remove the residual DNA, the RNA was sent to BGI (Beijing, China) where two cDNA libraries (PAB and PIB) were made and Solexa sequencing was performed. In all, 8,353,971 raw reads were obtained.

### Annotation and functional categorization

All clean reads were assembled by the standard SOAP *denovo* assembler ver 1.04^51^ (http://soap.genomics.org.cn/). The assembled contigs were subjected to a N50 algorithm^52^ to confirm the better performance of the assembly output. We defined such processed sequences as unigenes. In the final step, a Blastx alignment was performed using the unigenes and the NCBI non-redundant protein database (Nr), Swiss-Prot, KEGG, and COG protein databases^53^. The best-aligning results were used to determine the sequence directions of those unigenes. We then retrieved proteins that had the highest sequence similarity with the given unigenes and determined their functional annotations. We used the fatiGO and Blast2GO programs (http://www.blast2go.org/) to obtain the GO annotations for the unigenes^54^,^55^.

### Analysis of gene expressions by quantified real-time PCR

Total RNA was extracted from *Oncidium, Arabidopsis thaliana* ecotype Col-0, and *vtc1*. The mRNA in the total RNA (1 μg) was converted to first-strand cDNA using reverse transcriptase with the oligo-dT primer in accordance with the manufacturer’s instructions. qPCR was performed using Applied Biosystems 7500 Fast Real-Time PCR System and KAPA SYBR Green PCR^®^ master mixture universal. For qPCR analyses, gene expression levels were normalized based on the *Ubiquitin* gene of *Oncidium* or *Arabidopsis*. Sequence information for the oligonucleotides used for the qPCR analysis in this study is provided in Supplementary Table S3.

### NO detection

NO was quantified through DAF-2DA (Cayman) staining^56^. *Oncidium* PIB tissues at three different stages (V, B, and R), as well as *A. thaliana* Col and mutant seedlings were all stained according to the method described. Samples treated with elicitors were treated with CaCl_2_ for 1 h or H_2_O_2_ for 2 h and compared with mock at room temperature. Three biological replicates (3 biological replicates × 3 = 9 replicates per sample) of *Oncidium* PIB tissue at three different stages (V, B, and R) were used. For *Arabidopsis*, samples of more than twenty plants were used, and the averages of three biological replicates were tested. A two-way ANOVA analysis of variance was performed on the data to determine the significant differences and *p*-values utilizing GraphPad Prism® v5.0 software.

### Measurement of nitrate and nitrite reductase activities

*Oncidium* PLB and *Arabidopsis* leaves and roots were used to determine the nitrate reductase (NaR) and nitrite reductase (NiR) activity as previously described^57^,^58^. Samples (100 mg) were ground in liquid N_2_ and then homogenized in 0.5 mL of extraction buffer containing 3 mM EDTA and 50 mM Tris-HCl (pH 8.0). The mixture was centrifuged at 13,000 g for 20 min at 4 °C. For the NaR activity assay, the reaction buffer (250 μL of 0.1 M potassium-phosphate buffer (pH 7.5), 50 μL of 0.1 M KNO_3_ and 50 μL of 2 mM NADH) was added to the resulting supernatant (100 μL), and the mixture was incubated in the dark at room temperature for 60 min. The nitrate consumption was quantified at A_420_^59^. For the NiR activity assay, 550 μL of reaction buffer (500 μL of 0.1 M sodium phosphate buffer (pH 7.5), 25 μL of 10 mM NaNO_2_ and 50 μL of 1.5% methyl viologen) and 25 μL of 5% NaHCO_3_ were added to the resulting supernatant (100 μL), and the mixture was incubated at room temperature for 30 min. The reaction was stopped by the addition of the Griess reagent, and nitrite production was quantified at 540 nm^60^. The assay of nitrate concentration was determined based on forming a nitro derivative of salicylic acid with nitrate. Nitrite should be removed from the sample by saturated sulfamic acid. Samples and different concentrations of NaNO_3_ (nitrate standard) were extracted with a saturated solution of sulfamic acid. After incubating the mixture for 2 min, salicylic acid was added to reacts with nitrate under acidic conditions to form nitrosalicylic acid. Then the pH values were adjusted to 12 with NaOH. Nitrate concentration was determined after plotting against a linear regression and using spectrometry with A_420_^59^. The nitrite content was quantified from the NaR or NiR enzymatic assay described previously^61^. The averages of three biological replicates (3 technical replicates × 3 = 9 replicates per sample) from samples of orchid PLB tissues were used. For *Arabidopsis*, the average of the three biological replicates of more than twenty plant leaves and roots were used for the assay. A two-way ANOVA analysis of variance was performed on the data to determine the significant differences and *p*-values utilizing GraphPad Prism® v5.0 software.

### Quantification of arginine and citrulline by LC-ESI-MS

Samples were ground with a mortar and pestle in liquid N_2_, and metabolites were extracted in 80% methanol. Sample derivatization was performed with modifications^62^. In brief, 10 μL of metabolite samples was mixed with 10 μL of deionized water, 2.5 μL of 0.5 M Na_2_CO_3_ (pH 9.2), and 2.5 μL of 10 mg/μL dansyl chloride (freshly prepared in acetone), and the reaction was incubated at 60 °C for 1.5 h. Then, 75 μL of deionized water was added, and the mixture was further incubated at 60 °C for 30 min. Amino acid standards (physiological acids, neutrals and basics) (A9906; SIGMA) were prepared at various concentrations as described above for the derivatizing reagent and used to determine the concentrations of amino acids in the samples. The derivatizing samples were centrifuged at 14,000 rpm for 15 min, and the supernatants were subjected to LC-ESI-MS for arginine and citrulline quantification^63^. The average of three biological replicates (3 technical replicate × 3 = 9 replicates per sample) from samples of Orchid PLB tissues were used. For *Arabidopsis*, the average of three biological replicates of more than twenty plant leaves and roots were used for the assay. A two-way ANOVA analysis of variance was performed on the data to determine the significant differences and p-values utilizing Graphad prism v5.0 software^®^.

### Analysis of ascorbate and hydrogen peroxide

The extraction and measurement of AsA and H_2_O_2_ levels were performed as previously described^6^ with slight modification. *Oncidium* PLB tissues were washed with ddH_2_O twice. For total AsA quantification, the reactant was mixed with 10 mM dithiothreitol to reduce the pool of oxidized AsA. In contrast, to assay reduced AsA, only deionized water was added to the reactant. All mixtures were supplemented with reaction buffer (10% trichloroacetic acid, 43% H_3_PO_4_, 4% α-α-bipyridyl and 3% FeCl_3_) and incubated at 37 ± 1 °C for 1 h. The amounts of total and reduced AsA were determined by spectrometry at A_525_, and the amount of oxidized AsA was calculated as previously described. The average of three biological replicates (3 technical replicates × 3 = 9 replicates per sample) from samples of *Oncidium* PLB tissues was used, and two-way ANOVA analysis of variance was performed to determine the significant differences. The reduced and oxidized forms of AsA were determined from the total pool of three independent biological replicates.

## Additional Information

**Accession codes**: *AtNIA1* (AT1G77760), *AtNIA2* (AT1G37130), *AtNIR1* (AT2G15620) and *AtNOS/AtNOA1* (AT3G47450)

**How to cite this article**: Senthil Kumar, R. *et al*. Nitric oxide participates in plant flowering repression by ascorbate. *Sci. Rep.* **6**, 35246; doi: 10.1038/srep35246 (2016).

## Supplementary Material

Supplementary Information
