# Supplementary material for: Nitric oxide participates in plant flowering repression by ascorbate
Source: Sci Rep. 2016 Oct 12;6:35246. doi: 10.1038/srep35246 (PMC5059679; doi:10.1038/srep35246)

**Nitric oxide participates in plant flowering repression by ascorbate**

^§1^Rajendran Senthil Kumar,^§1^Chin-Hui Shen, ^1^Pei-Yin Wu, ^1^Suresh Kumar, ^1^Moda Sang Hua, and ^1^Kai-Wun Yeh*

**Supplementary Table S1** Statistical characterization of contigs generated from *Oncidium*

Summary PIB PAB

**Output Statistics**

Total number of reads 26,666,668 26,666,668

Total nucleotides (nt) 2,400,000,120 2,400,000,120

GC percentage 47.72% 46.36%

Q20 percentage 93.39% 93.92%

**Assembly results**

Total number of contigs 665,127 925,937

Average sequence size of contigs 119 115

Total number of scaffolds 170,137 214,399

Average sequence size of scaffolds 227 215

Total number of unigenes 92,989 114,521

Total nucleotides (nt) in unigenes 29,351,181 34,133,757

Average sequence size of unigenes 316 298

**Annotation results combined three libraries**

Total number of afresh-assembled unigenes 108,551

Average sequence size of afresh-assembled unigenes 409

Number and percentage of annotated

unigenes (Nr/Swiss-Prot) 51,883/32,747(47.8%/30.2%)

Number and percentage of annotated

unigenes (KEGG/COG) 21,781/14,259(20.1%/13.1%)

PAB: pseudobulb with axillary bud; PIB: pseudobulb with inflorescent bud

**Supplementary Table S2 The pathway enrichment analysis of PAB library versus PIB library**

| GO category | GO subcategory (term number) | supported category (term number) |
| --- | --- | --- |
| Biological processes  Molecular functions  Cellular components | cellular process (GO: 0009987)  metabolic process (GO:0008152)  catalytic activity (GO: 0003824)  binding(GO: 0005488)  organelle (GO: 0044422)  cell (GO: 00044464) | ribosome biogenesis (GO: 0042254)  chromatin assembly or disassembly (GO: 0006333)  cell redox homeostasis (GO: 0045454)  gluconeogenesis (GO: 0006094)  ATP biosynthetic process (GO: 0006754)  GTPase activity (GO: 0003924  hydrogen ion transmembrane transporter activity (GO: 0015078)  GTP binding (GO: 0005525)  peptide binding (GO: 0042277)  magnesium ion binding (GO: 0000287)  nucleic acid binding (GO: 0003676)  chromatin binding (GO: 0003682)  mitochondrial inner membrane (GO: 0005743)  ribosomal subunit (GO: 0033279) |

PAB: pseudobulb with axillary bud; PIB: pseudobulb with inflorescent bud

**Table S3.** Lists of primers for Qpcr

Primer name (GeneBank No. or Unigene No.) sequence

*OgHb-1*(Unigene96319)_FP 5-ATGACCTCTAATGGCTCAGGA-3

*OgHb-1*(Unigene96319)_RP 5-TGGAACCTTCGAGTCTCGCA-3

*OgMPT*(Unigene659)_FP 5-ACTTCCTCTCTCTGCCCAT-3

*OgMPT*(Unigene659)_RP 5-TCCCCGTCCTCATAAACCTC-3

*OgNaR*(Unigene4706)_FP 5-TGGTGCTGGTGTTTCTGGTC-3

*OgNaR*(Unigene4706)_RP 5-TGGGTTGGGTGTTGGAAAGA-3

*OgNaT*(Unigene28206)_FP 5-CCTGGTAGGAGCATCAGAGG-3

*OgNaT*(Unigene28206)_RP 5-AATCCACCCAGGTCTGTCGT-3

*OgNiR*(Unigene98089)_FP 5-AGCTCCCTGATGTCCCCTCA-3

*OgNiR*(Unigene98089)_RP 5-TTTGAATTGGCGGTGACGAA-3

*OgNiT*(Unigene104905)_FP 5-ATCCGACATCACAACCACCA-3

*OgNiT*(Unigene104905)_RP 5-ATGGATTTGGCTTGGATGAT-3

*OgNOS*(Unigene53152)_FP 5-ACCTGCATCCGACATAGCGA-3

*OgNOS*(Unigene53152)_RP 5-GTACCACTTAGGTCTCAGCTCTTC-3

*OgNOSAP*(Unigene20914)_FP 5-TCAAAGTCGTCTCAGGGGAC-3

*OgNOSAP*(Unigene20914)_RP 5-TGGGACCAACAGAATTTGCA-3

*OgNOSIP*(Unigene42661)_FP 5-AGCGGCACTCGAAGAACAA-3

*OgNOSIP*(Unigene42661)_RP 5-CTGGGCAGCATGGACAGCTA-3

*OgNOSPI*(Unigene36414)_FP 5-ATGAGGTGCAGAAGGAGGCT-3

*OgNOSPI*(Unigene36414)_RP 5-GAAGTGGTTTGTCTCATGGGTA-3

*OgFT*(ACC59806)_FP 5-ACCTCAGGACTTTCTACACTCTTG-3

*OgFT*(ACC59806)_RP 5-GAAACAGCACGAACACGAAGC-3

*OgFYF*(ADU17781)_FP 5-GTTCTGCTGAAGAGCTCCATGA-3

*OgFYF*(ADU17781)_RP 5-GGCCTTCTCAGGCTTCTTACAC-3

*OgAP1*(HM140844)_FP 5-GCTCCTCGCCTCCTTCCTA-3

*OgAP1*(HM140844)_RP 5-TACTCACCGGGTATGTCCCTAAA-3

*OgTFL1*(HQ832786)_FP 5-TGTAGTTGGTAGAGTTATAGGAGAAG-3

*OgTFL1*(HQ832786)_RP 5-ATCAGTCATAATCAGTGTGAAGAAAG-3

*AtNIA1*(AT1G77760)_FP 5-GTGGTTTACGCAAACAGAACCGAGG-3

*AtNIA1*(AT1G77760)_RP 5-TTCGCCTTCCAAACCTTCAGGGAT-3

*AtNIA2*(AT1G37130)_FP 5-TTGGGCAGAGCAATACCCGGA-3

*AtNIA2*(AT1G37130)_RP 5-TCGGCTGAACCGCAAACTGAATCA-3

*AtNIR1*(AT2G15620)_FP 5-TAGCCAGTTCTGCGGACAAGCGA-3

*AtNIR1*(AT2G15620)_RP 5-ACGTCAGCACCCTCGACTGGCT-3

*AtNOS/AtNOA1*(AT3G47450)_FP 5-ACGAGGCAAAACTGGGTGTGCTTC-3

*AtNOS/AtNOA1*(AT3G47450)_RP 5-TCCTCGTGTTTTGCGGATTGGTTC-3


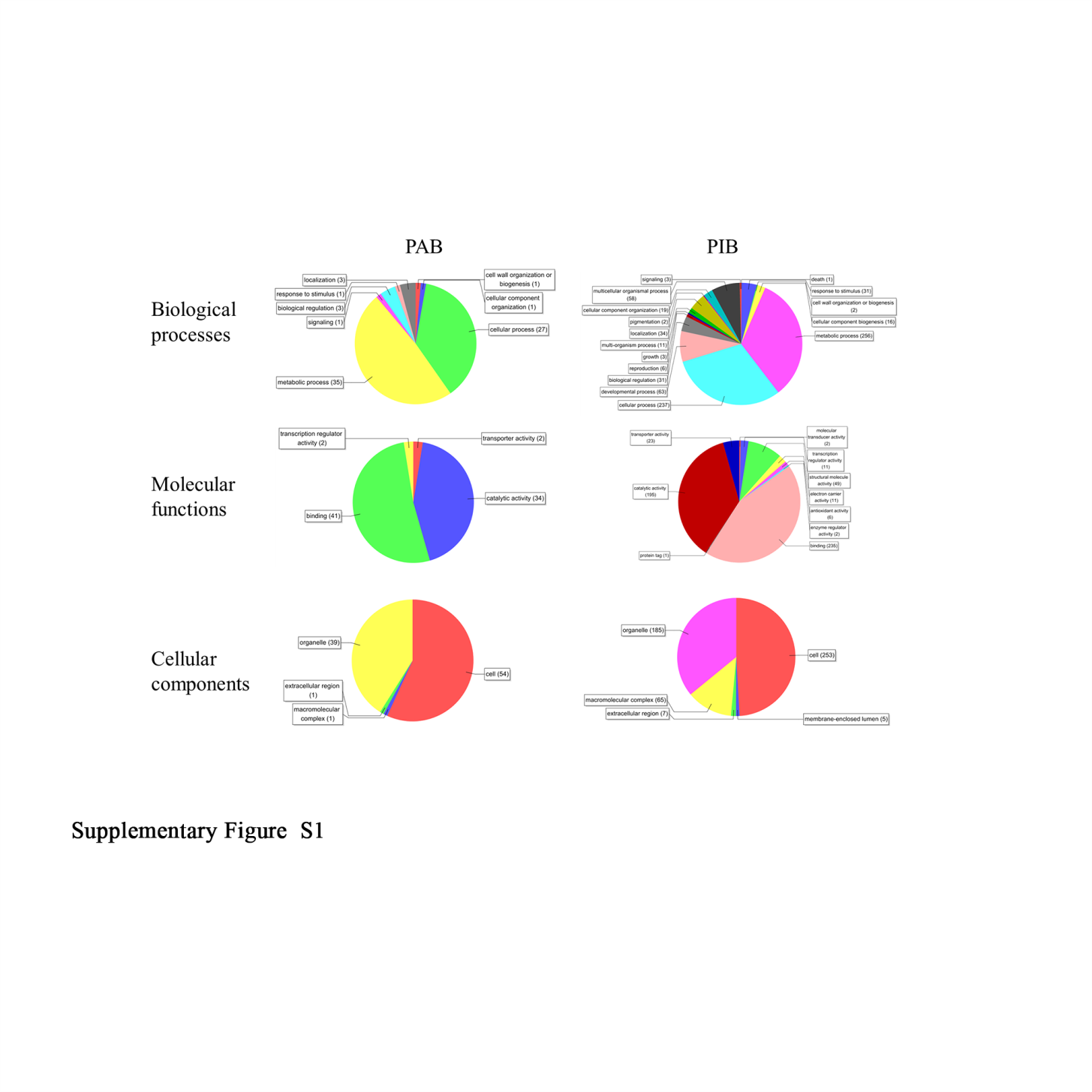


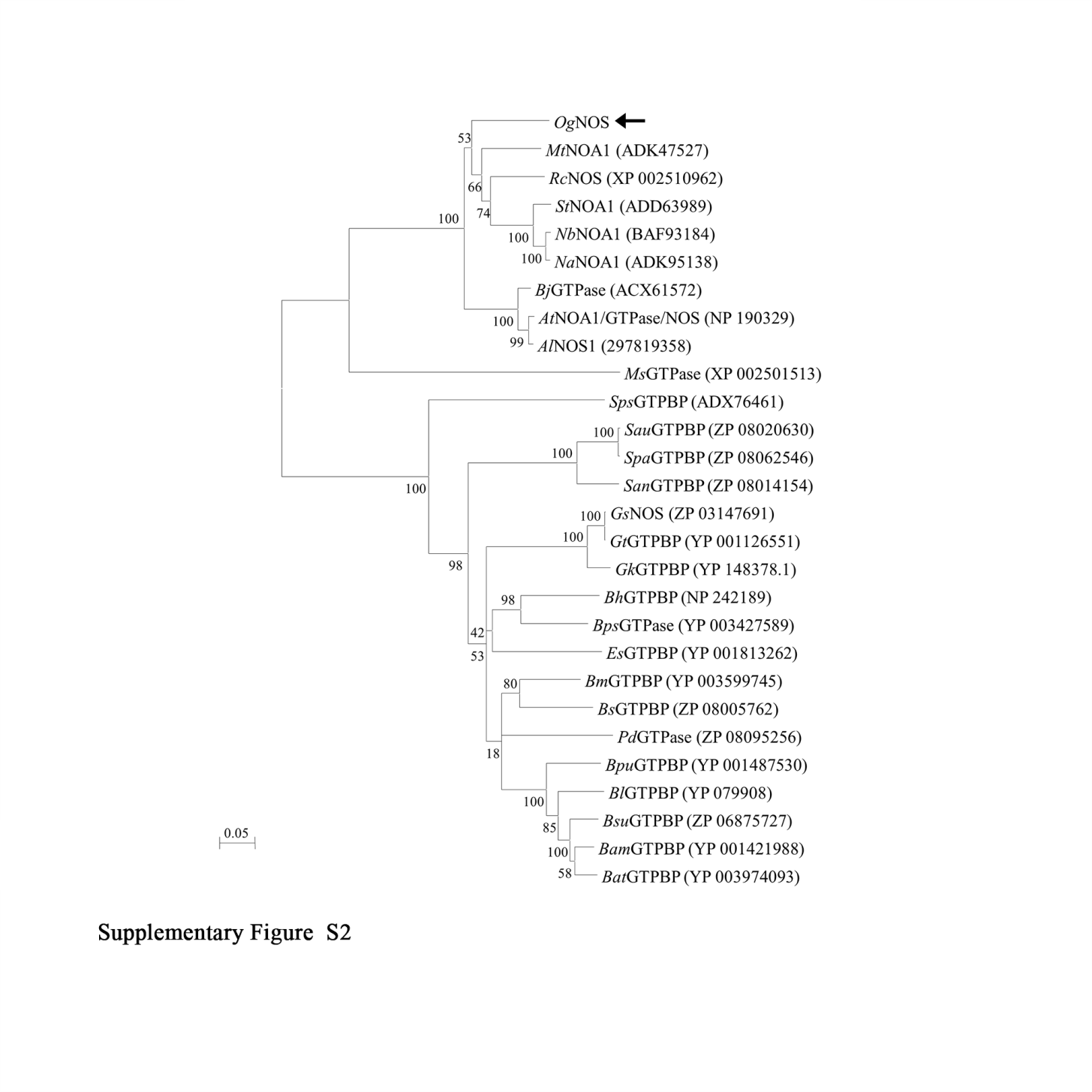


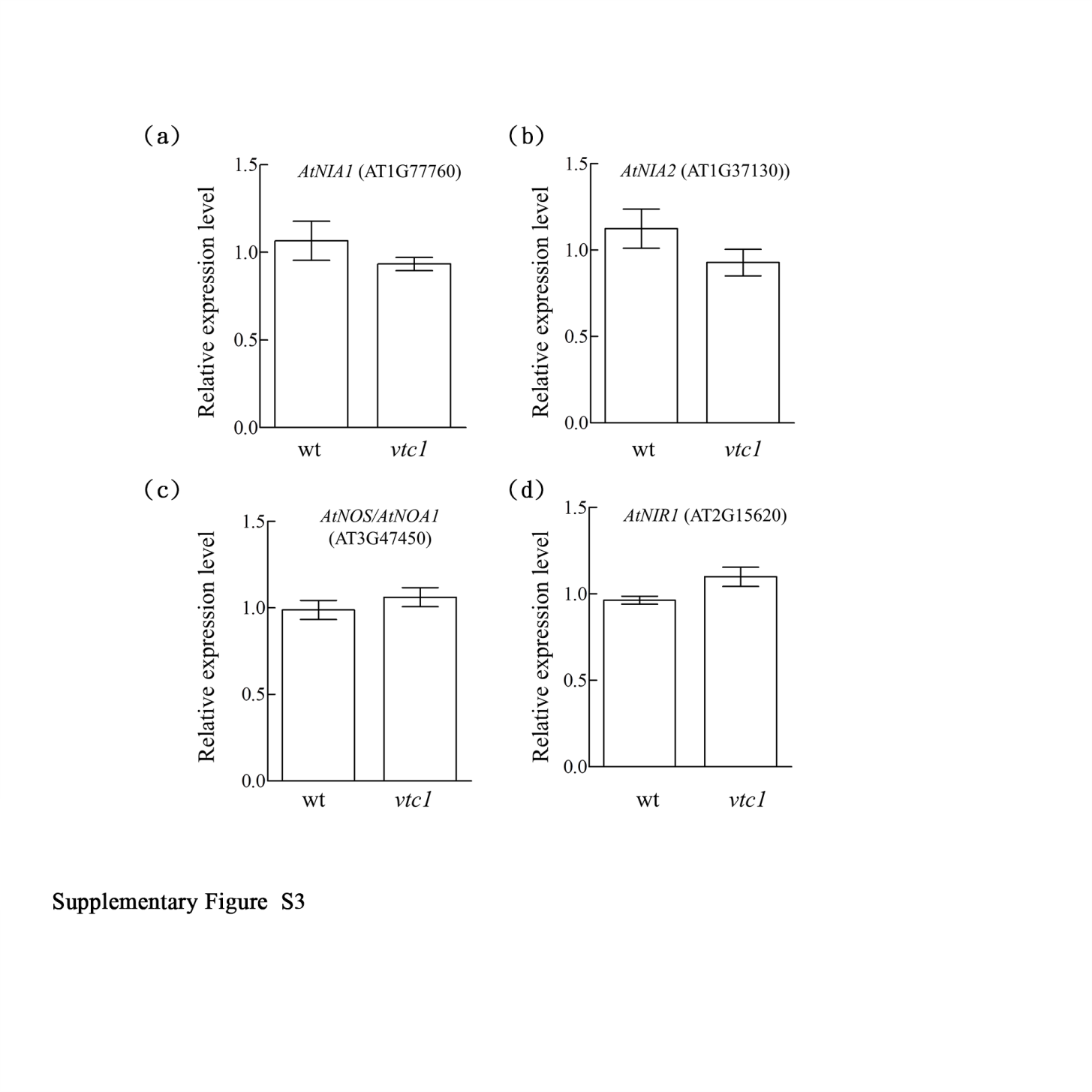

Supplement: Supplementary Information [file srep35246-s1.doc]
